# A dataset of proteomic changes during human heat stress and heat acclimation

**DOI:** 10.1038/s41597-023-02809-5

**Published:** 2023-12-07

**Authors:** Daniel Gagnon, Hadiatou Barry, Amina Barhdadi, Essaid Oussaid, Ian Mongrain, Louis-Philippe Lemieux Perreault, Marie-Pierre Dubé

**Affiliations:** 1 Montreal Heart Institute, Montreal, QC Canada; 2 School of Kinesiology and Exercise Science, Université de Montréal, Montreal, QC Canada; 3 Department of Pharmacology and Physiology, Université de Montréal, Montreal, QC Canada; 4 Université de Montréal Beaulieu-Saucier Pharmacogenomics Centre, Montreal, QC Canada; 5 Department of Medicine and Department of Social and Preventive Medicine, Université de Montréal, Montreal, QC Canada

**Keywords:** Protein-protein interaction networks, Neurophysiology

## Abstract

Hotter climates have important impacts on human health and performance. Yet, the cellular and molecular responses involved in human heat stress and acclimation remain understudied. This dataset includes physiological measurements and the plasma concentration of 2,938 proteins collected from 10 healthy adults, before and during passive heat stress that was performed both prior to and after a 7-day heat acclimation protocol. Physiological measurements included body temperatures, sweat rate, cutaneous vascular conductance, blood pressure, and skin sympathetic nerve activity. The proteomic dataset was generated using the Olink Explore 3072 assay, enabling a high-multiplex antibody-based assessment of protein changes based on proximity extension assay technology. The data need to be interpreted in the context of the moderate level of body hyperthermia attained and the specific demographic of young, healthy adults. We have made this dataset publicly available to facilitate research into the cellular and molecular mechanisms involved in human heat stress and acclimation, crucial for addressing the health and performance challenges posed by rising temperatures.

## Background & Summary

Climate change is increasing the occurrence of heat extremes, reaching intensities that may surpass the limits of human adaptability by the end the century^1^. To thrive in a climate marked by more frequent heat extremes, humans must adapt both behaviorally and physiologically^2^. Humans have demonstrated a physiological capability to adapt to recurring heat exposure, through changes in thermoregulatory and cardiovascular responses^3^. However, the cellular and molecular mechanisms mediating human heat adaptation remain understudied.

Our current understanding of the cellular and molecular mechanisms that mediate human heat adaptation relies upon studies performed on rodents. These studies have shown that repeated exposures to heat stress performed over a relatively short timeframe (days to weeks, i.e., heat acclimation) result in gene expression changes related to the heat shock response, as well as anti-apoptotic and anti-oxidative networks that improve cellular resilience, ultimately leading to survival under severe levels of hyperthermia (core temperature ≥40 °C)^4^. In humans, studies have shown that heat stress can up or down regulate gene expression involved in various biological processes including the heat shock response, energy metabolism, proteostasis, immune responses, cell growth and proliferation, as well as cell death and survival^5–8^. One limitation of these studies is that the analyses were limited to gene expression changes that occur in response to heat stroke^5–7^ or very brief (≤15 min) heat exposure^8^. Although severe hyperthermia contributes to a greater incidence of heat stroke during heat extremes, most adverse heat-related outcomes are attributed to moderate hyperthermia and the resulting physiological strain^9^. Additionally, human heat acclimation occurs as a result of repeated exposures to moderate, rather than severe, hyperthermia^3^. Furthermore, only a few studies have examined how gene expression changes translate into changes at the protein level. These studies mainly targeted heat shock proteins and have produced inconsistent results^10^. Therefore, our current understanding of the cellular and molecular responses to heat exposure and acclimation in humans is mainly based on studies investigating changes in gene expression or heat shock proteins in response to severe levels of hyperthermia that do not accurately represent the levels of heat strain that humans will increasingly experience due to climate change.

To further our understanding of the biological responses and adaptations to heat exposure, we performed an untargeted, large-scale proteomic analysis to determine how plasma protein concentrations change in response to moderate hyperthermia and heat acclimation. We reasoned that such an untargeted analysis would promote research into previously unexplored proteins that may be involved in the human body’s response and acclimation to heat exposure. The data are made publicly available with the aim to promote their broad use, including by scientists from diverse disciplines.

**Fig. 1:**
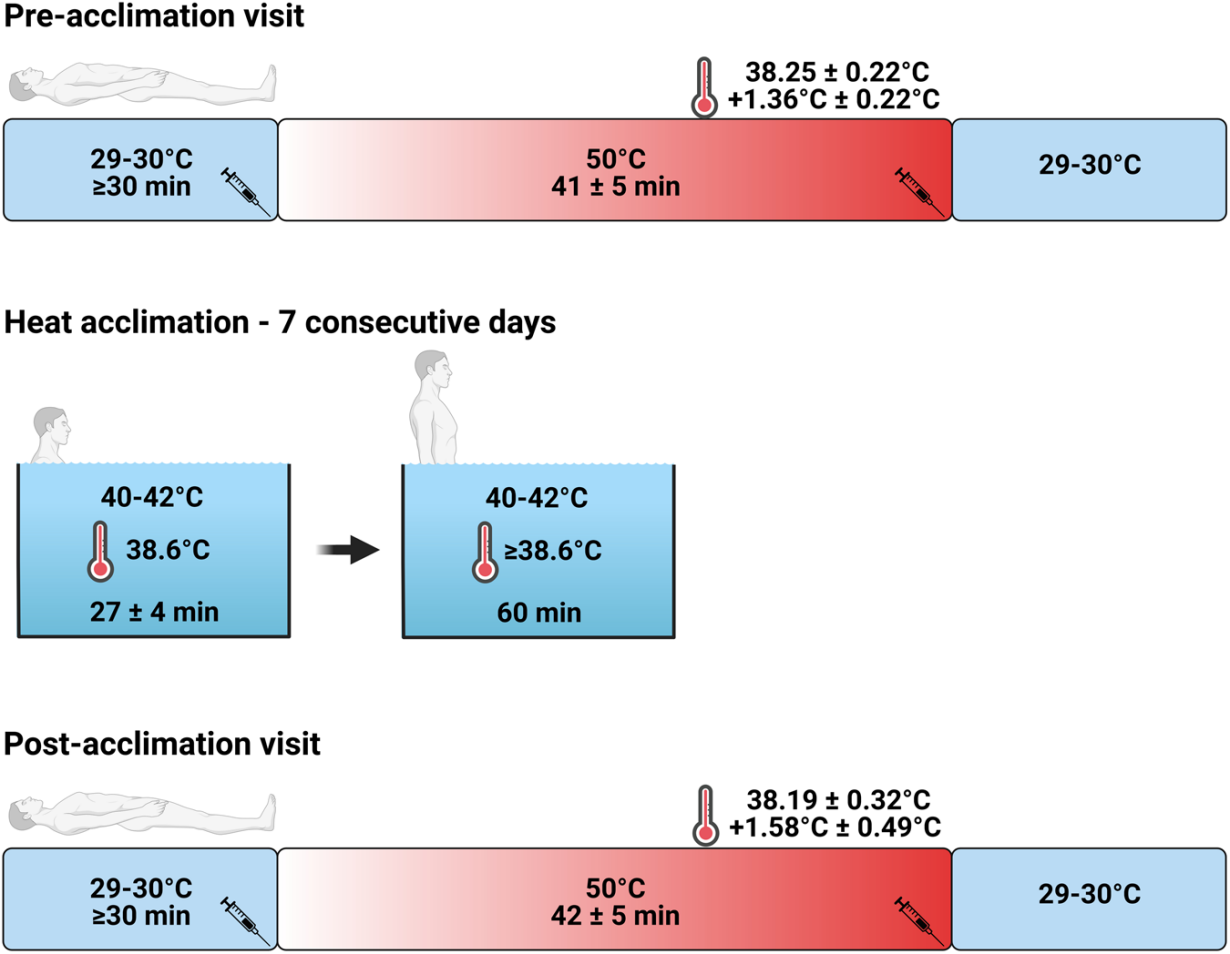
Overview of the study design. Participants were exposed to passive heat stress with a water-perfused suit before and after heat acclimation. During the pre-acclimation visit, participants were heated until core temperature increased ~1.4 °C. During the post-acclimation visit, participants were heated until core temperature attained the same absolute value that was reached during the pre-acclimation visit (~38.2 °C). Blood samples were drawn before and at the end of heat stress for plasma proteomic analyses. The heat acclimation protocol involved hot water immersion at shoulder level until core temperature reached 38.6 °C, after which participants sat within the water bath to maintain core temperature ≥38.6 °C for 60 minutes.

The data and samples used for this proteomics dataset were collected as part of a larger study that studied physiological adaptations to heat acclimation. The study involved two laboratory visits performed before and after a 7-day heat acclimation protocol (Fig. 1). The physiological data have been published previously^11–13^. In this manuscript, we present the proteomics dataset that was generated from blood samples collected on a sub-group of 10 healthy adults, 4 females and 6 males, with a mean age of 25 ± 3 years and a mean body mass index of 24.5 ± 2.7 kg/m^2^. Proteomic analyses were performed with the Olink Explore 3072 assay, a high-multiplex immunoassay platform that uses Proximity Extension Assay (PEA) technology based on DNA sequencing for high-throughput readout. The data should be interpreted in the context of the moderate level of hyperthermia that was elicited during heat stress, as well as the population studied, which consisted of young and healthy adults.

The large number of proteins analyzed in this study will support future investigations. The individual protein results can be utilized as external validation for other studies, as the basis of further data analysis explorations, and/or to guide targeted investigations of the biological responses and adaptations to heat exposure.

## Methods

### Ethical approval

The study was approved by the Research Ethics and New Technologies Development Committee of the Montreal Heart Institute (approval #2016–2083). All participants provided verbal and written informed consent. The study conformed to the standard set by the Declaration of Helsinki, except for registration in a database.

### Participants

As previously described, participants were eligible to take part in the study if they were aged between 18 and 40 years^11^. Exclusion criteria included a body mass index  ≥30 kg/m^2^, current smoking, diagnosis of chronic disease, prescription of medications, an abnormal resting electrocardiogram (ECG), a resting blood pressure greater than 150/90 mmHg, a resting heart rate greater than 100 beats/min, pregnancy or breast-feeding for female participants. Before each visit, participants were instructed to consume ~500 mL of water and eat a light snack the morning of each visit, and abstain from caffeine, alcohol, and strenuous exercise for at least 12 hours. Hydration status was measured upon arrival for each laboratory visit using urine specific gravity. If urine specific gravity was greater than 1.025, participants were asked to drink 250 mL of water before continuing with the visit.

### Laboratory visits

All visits began in the morning between 8:00 AM and 9:00 AM. Upon arrival, participants emptied their bladder and weighed themselves nude. They then wore a water-perfused suit (COOLTUBEsuit, Med-Eng, LLC) and rested supine while 29–30 °C water was circulated (AD07H200, PolyScience) through the suit for a minimum of 30 minutes. During this time, participants were fitted with a sweat capsule and a laser-Doppler flow probe on the dorsal side of the forearm to measure heat loss thermoeffector responses. A microelectrode was also inserted into the radial nerve to measure skin sympathetic nerve activity. Once instrumentation was completed, participants rested for 10 minutes and a blood sample was drawn for the normothermic measurement before the temperature of the water perfusing the suit was increased to 50 °C and maintained at this temperature until core temperature increased by ~1.4 °C above baseline values (duration of 2448 ± 313 s). During the post-acclimation visit, heat exposure continued until the core temperature reached the same absolute temperature achieved during the pre-acclimation visit (~38.2 °C, duration of 2490 ± 285 s). A blood sample was taken during the last 5 minutes of heating for the hyperthermic measurement, after which the water perfusing the suit was reduced to 15 °C to cool the participant. The participant was then de-instrumented and a final nude body weight was measured. Fluid intake was not permitted during heat exposure. The pre-acclimation visit was conducted within 7 days of beginning the heat acclimation protocol. The post-acclimation visit was performed within 12–48 h of completing the acclimation period.

### Heat acclimation

Heat acclimation was achieved through a 7-day protocol that involved controlled hyperthermia^14^. Participants were initially immersed to shoulder level in a circulated water bath (S-110-M, Whitehall Manufacturing) maintained at 40–421 °C by an external heating unit (Compact dual temp, iCool) until their core temperature reached 38.6 °C (~27 minutes). Participants then sat on a bench placed within the bath so that water was at waist-level to maintain their core temperature ≥ 38.6 °C for an additional 60 minutes. Participants could drink water and/or an electrolyte beverage (Power Quencher 2, President Choice: 591 ml/bottle, Sodium 260 mg, Potassium 90 mg, Carbohydrate 13 g) *ad libitum* during the visits. Participants were instructed to maintain their lifestyle habits during the acclimation period, but to avoid strenuous exercise for 12 h before each visit.

### Instrumentation

Core temperature was measured in the esophagus (pre/post visits) or rectum (heat acclimation visits) using pediatric grade thermistors (400 series, 9Fr, Covidien). Mean skin temperature was calculated as a weighted average of measurements on the arm, chest, thigh, and calf^15^ using t-type thermocouple wire (EXPP-T-20-SLE-500, pre/post visits) or wireless temperature sensors (iButtons DS1922L-F5, Embedded Data Systems, LLC, acclimation visits). Forearm sweat rate was measured by ventilating dry compressed air (600 mL/min) through a plastic capsule (2.83 cm^2^) and measuring the absolute humidity of the effluent air with a factory-calibrated capacitance hygrometer (HMT130, Vaisala Canada Inc.). Forearm skin perfusion was measured using a laser-Doppler probe (VP7A/T, Moor Instruments). Skin sympathetic nerve activity was measured by inserting a tungsten microelectrode (UNAF2T, FHC Inc.) into the radial nerve under ultrasound (uSmart 3300, Terason) guidance^16^. Forearm sweat rate and skin perfusion were measured on the same arm from which skin sympathetic nerve activity was measured. Nude body weight was measured with a high-performance digital scale (IND236, Mettler-Toledo, precision: 0.01 kg). Heart Rate was calculated from the R-R interval of a 5-lead ECG signal (Solar i8000, GE Healthcare). Systolic and diastolic blood pressure was measured using an automated ECG-gated monitor (Tango M2, SunTech Medical). Urine specific gravity was measured using a digital refractometer (PAL-10S, Atago).

### Proteomic data measurements

Blood samples were drawn into a vacutainer containing a 3.2% sodium citrate solution (BD Biosciences) and immediately centrifuged (1000 g for 15 min) to aliquot and store plasma at −80 °C. Forty frozen citrate plasma samples were shipped to the Analysis Service unit at Olink Proteomics in Boston, USA, where proteomic analyses were performed with all samples distributed onto a single plate. The Explore 3072 assay was conducted by using two separate assays, the Olink Explore 1546 (results issued on 2021-11-30) and the Explore Expansion assay (results issued on 2021-12-13). Proximity Extension Assay (PEA) technology was conducted according to the Olink AB manufacturer procedures by the certified laboratory. Briefly, the technique relies on the use of antibodies labelled with unique DNA oligonucleotides which bind to a target protein. When in proximity on a target protein, the DNA oligonucleotides hybridize and serve as a template for DNA polymerase-dependent extension, creating a unique double-stranded DNA barcode proportional to the initial protein concentration. The resulting DNA amplicons are then quantified using high throughput DNA sequencing on the Illumina NovaSeq platform. Reported protein measurements are based on the Normalized Protein eXpression (NPX) values, a relative protein quantification unit, normalized to control for systematic noise caused by sample processing and technical variation based on internal controls and based on sample controls (https://www.olink.com/content/uploads/2021/09/olink-data-normalization-white-paper-v2.0.pdf). NPX units are on a log2 scale, where an increase by one NPX unit corresponds to a two-fold increase in the concentration of the amplicons representing the target protein compared to the internal control. Data values that passed quality control (QC) with measurements below the limit of detection (LOD) were included. Data files containing results were securely transferred from the Service laboratory to the research team.

### Physiological data measurements

Continuous data were recorded at 1000 Hz (PowerLab 16/35, AD Instruments Inc.). Mean body temperature was calculated using a weighted average of core temperature (0.8) and mean skin temperature (0.2)^11^. Forearm sweat rate was calculated by multiplying the effluent humidity from the ventilated capsule by flow rate and dividing by the surface area covered by the capsule. Cutaneous vascular conductance, an index of cutaneous vasodilation^17^, was calculated as the skin perfusion units divided by the mean arterial blood pressure. Mean arterial blood pressure was calculated as the sum of the diastolic blood pressure and 1/3 of the pulse pressure. Whole-body sweat rate was calculated from changes in nude body weight (accounting for any fluid consumption and/or urine loss) divided by the time separating each weight measurement. Skin sympathetic nerve activity was quantified by integrating the area beneath each individual burst during a 3-minute recording at baseline and a 1-minute recording at the end of heat stress^11^. The value during heat stress was expressed as a percentage change from the baseline value, to account for variability in the location of the microelectrode within the nerve bundle which cannot be controlled^18,19^.

## Data Records

The data are openly available as Figshare project 163291: https://figshare.com/projects/Proteomic_changes_during_human_heat_stress_and_heat_acclimation/163291.

### Physiological measurements

The physiological measurements made on the participants during the pre and post acclimation visits are presented as an Excel file (named “Physiological data”)^20^. The Excel file includes one sheet (named “Heat exposure”) that contains the data collected during the normothermic (“initial”) and hyperthermic (“final”) measurements, both before and after heat acclimation, including: core temperature (Tcore), mean skin temperature (Tskin), mean body temperature (Tbody), local sweat rate (LSR), laser-Doppler flux values (LDF), cutaneous vascular conductance (CVC), heart rate (HR), systolic blood pressure (SBP), diastolic blood pressure (DBP), mean arterial blood pressure (MAP), skin sympathetic nerve activity (SSNA), the change in nude body weight (post minus pre heat exposure), heat exposure duration, and whole-body sweat rate.

### Proteomics data

The proteomic data received from the Olink service provider is made available (named “Raw Olink data”)^21^. The zip archive contains two files, one for the Olink Explore 1546 and one for the Explore Expansion assay results. The files contain the results for the 10 study participants at 4 timepoints. The fields are separated by a semicolon. The sample IDs contains information about the sample and the timepoints. For example, sample “SSNA-001B-PR1” represents the normothermic, pre-acclimation NPX data for sample 001B. The abbreviations used for the four different timepoints are PR1: normothermic, pre-acclimation; PT1: hyperthermic, pre-acclimation; PR2: normothermic, post-acclimation; PT2: hyperthermic, post-acclimation. In total, for each sample, there are 2,943 assays (separated into 2 files) for each of the 4 timepoints. Note that some proteins are duplicated across panels, for example, protein TNF (UniProt P01375) is included in 4 of the 8 panels, so the OlinkIDs should be used as unique identifiers for the proteins. The file “20212016_Dube_NPX_2021-11-30.csv” contains NPX data for 1,472 assays of the Olink Explore 1546 panel divided into 4 panels: cardiometabolic, inflammation, oncology and neurology. The file “20212017_Dube_NPX_2021-12-13_OID30253_corrected.csv” contains NPX data for the 1,471 assays of the Explore Expansion panel divided into 4 additional panels: cardiometabolic II, inflammation II, oncology II and neurology II. Note that the data in the file include a correction to Olink assay ID (OID30253), which was flagged by the service provider. The incorrect OID30253 had UniProtID A0A0B4J2D5 and gene GATD3B, while the corrected OID30253 has UniProtID P0DPI2 and gene GATD3. This assay is part of the Cardiometabolic II panel, and the correction was applied to the file.

In addition, the proteomic NPX values were filtered according to Quality Control (QC) criteria and are made available (named “Filtered NPX values”)^22^. The dataset contains the results for all 10 participants at the 4 timepoints distributed as one file for each of the 8 Olink panels. The filtering process consisted of setting NPX values as missing when either the “QC_Warning” or “Assay_Warning” fields that were not flagged as “PASS” by the QC procedure.

### Changes in NPX values

The change (delta) in NPX values for all proteins measured per participant is provided in the “Changes in NPX values” zip archive, including one CSV file for each of the four change measurements considered^23^. The change measurements include “dNPX_accpost” representing the effect of acclimation measured in the hyperthermic state, generated by subtracting the hyperthermic pre-acclimation NPX values from the hyperthermic post-acclimation NPX values. The change measurement “dNPX_accpre” represents the effect of acclimation in the normothermic state, generated by subtracting the normothermic pre-acclimation NPX values from the normothermic post-acclimation NPX values. The “dNPX_hd1” measurement represents the effect of heat stress prior to acclimation, generated by subtracting the normothermic pre-acclimation NPX values from the hyperthermic pre-acclimation NPX values. Finally, the “dNPX_hd7” measurement represents the effect of heat stress after acclimation, it was generated by subtracting the normothermic post-acclimation NPX values from the hyperthermic, post-acclimation NPX values.

### Fold change in protein levels

The log2 (fold change) in the level of each protein is provided in a zip archive that contains one file for each of the 8 Olink panels (named “Fold Change (log2)”)^24^. The files include log2 fold change values for the 4 different change measurements described below.

### Proteomic changes pre-acclimation

Fold change in protein levels from normothermic pre-acclimation to hyperthermic pre-acclimation (under the column heading “PT1-PR1”) are provided in the files.

### Proteomic changes post-acclimation

Fold change in protein levels from hyperthermic pre-acclimation to hyperthermic post-acclimation (“PT2-PT1”), from normothermic pre-acclimation to normothermic post-acclimation (“PR2-PR1”) and from normothermic post-acclimation to hyperthermic post-acclimation (“PT2-PR2”) are provided.

## Technical Validation

For the Olink proteomic assay, the 40 samples (taken at 4 timepoints for 10 participants) were randomly distributed onto a single plate to conduct the analyses. The proteomic assays were conducted using 3 internal controls added to each sample, including an expansion control used for the generation of the NPX values, an incubation control used to monitor the quality of assay performance and an amplification control to monitor sample quality. In addition, three external controls are included, including a plate control (healthy pooled plasma) used for data normalization, a sample control used to assess variation between runs, and a negative control used to calculate the limit of detection for each assay and to assess potential contamination. To pass QC, there had to be at least 500 sequencing reads per sample, the deviation from the median value of the incubation and amplification controls for each individual sample had to not exceed +/−0.3 NPX for either of the internal controls, and the deviation of the median value of the negative controls had to be ≤5 standard deviations from the set predefined value.

There were 5 protein assays out of 2,943 that failed to pass QC and were removed from the filtered dataset. Out of 117,520 individual sample assays conducted, 3,515 (3.0%) failed to pass QC. All samples that did not pass QC criteria were excluded from analyses. There were 864 (29.4%) proteins with missing data in more than 20/40 samples out of the 2,938 protein assays included in the analyses. Missing data were not imputed. Documents with details of the validation results for each individual assay included in the Explore 3072 are available from Olink Proteomics AB (https://olink.com/resources-support/document-download-center).

## Data Availability

The source code for this dataset is publicly available through the Figshare repository^25^. The data may also be visualized with an interactive web visualization tool: https://acclimation.statgen.org/
